# Genetic Characterization of Hepatitis C Virus Among People Who Use Crack Cocaine: A Study Conducted on the Brazilian Amazon Coast

**DOI:** 10.3390/pathogens14121296

**Published:** 2025-12-17

**Authors:** João Alphonse A. Heymbeeck, Wilker Leite do Nascimento, Marina Cristina S. Freitas, Leticia de Sousa Rocha, Franciane Ferreira Costa, Jocilena Pamela Q. de Queiroz, Diego Simeone, Luísa Caricio Martins, Luiz Fernando A. Machado, Benedikt Fischer, Emil Kupek, Aldemir B. Oliveira-Filho

**Affiliations:** 1 Residência Multiprofissional de Saúde da Mulher e da Criança, Universidade Federal do Pará, Bragança 68600-000, PA, Brazil; joao.heymbeeck@gmail.com (J.A.A.H.); losenwil@gmail.com (W.L.d.N.); caricio@ufpa.br (L.C.M.); 2 Grupo de Estudo e Pesquisa em Populações Vulneráveis, Instituto de Estudos Costeiros, Universidade Federal do Pará, Bragança 68600-000, PA, Brazil; marinacristina51@gmail.com (M.C.S.F.); leticiarochalele@gmail.com (L.d.S.R.); francycostta8@gmail.com (F.F.C.); jocilenaqueiroz85@gmail.com (J.P.Q.d.Q.); 3 Programa de Pós-Graduação em Doenças Tropicais, Universidade Federal do Pará, Belém 66055-240, PA, Brazil; 4 Programa de Pós-Graduação em Biologia Ambiental, Universidade Federal do Pará, Bragança 68600-000, PA, Brazil; 5 Afya Faculdade de Ciências Médicas, Bragança 68600-000, PA, Brazil; diegosimeone.bio@gmail.com; 6 Núcleo de Medicina Tropical, Universidade Federal do Pará, Belém 66055-240, PA, Brazil; 7 Instituto de Ciências Biológicas, Universidade Federal do Pará, Belém 66075-110, PA, Brazil; lfam@ufpa.br; 8 Faculty of Health Sciences, Simon Fraser University, Vancouver, BC V6B 5K3, Canada; bfischer@sfu.ca; 9 Waypoint Research Institute, Waypoint Centre for Mental Health Care, Penetanguishene, ON L9M 1G3, Canada; 10 Research & Graduate Studies, University of the Fraser Valley, Abbotsford, BC V2S 7M8, Canada; 11 Department of Psychiatry, University of Toronto, Toronto, ON M5T 1R8, Canada; 12 School of Population Health, Faculty of Medical & Health Sciences, Grafton Campus, University of Auckland, Auckland 1023, New Zealand; 13 Department of Psychiatry, Federal University of São Paulo, São Paulo 04017-030, SP, Brazil; 14 Departamento de Saúde Pública, Universidade Federal de Santa Catarina, Florianópolis 88040-900, SC, Brazil; emil.kupek@ufsc.br

**Keywords:** viral hepatitis, genotypes, resistance-associated substitutions, key populations, health policies

## Abstract

People who use crack cocaine (PWUCC) constitute a key population due to vulnerability and marginalization, especially in a socio-ecologically diverse, relatively isolated region with limited public health infrastructure. This study aimed to perform a genetic characterization of circulating HCV among PWUCC in the municipality of Bragança, situated on the Brazilian Amazon coast, identifying viral genotypes, subtypes, resistance-associated substitutions (RAS)—naturally occurring mutations in the viral genome that can reduce the efficacy of direct-acting antiviral (DAA) agents—and predictions of phenotypic resistance. Methods: Between 2016 and 2018, biological samples and epidemiological data were obtained from 165 PWUCC. Viral detection was performed using RT-PCR, while genotyping, subtyping, and RAS profiling were conducted through nucleotide sequencing and fragment analysis. Results: In 165 PWUCC, 22 (13.3%) tested positive for HCV RNA. Most of them had not had access to public health services (91.5%), and more than half (57.0%) reported living in unstable housing conditions. HCV subtypes 1a (27.3%), 1b (40.9%), and 3a (31.8%) were detected. Evidence of resistance associated with DAAs, such as daclatasvir and dasabuvir, was detected in five PWUCC with HCV (22.7%). Conclusions: The high prevalence of HCV infection, predominantly subtype 1b, and significant levels of resistance are very concerning. This demonstrates the urgent need for targeted public health interventions to expand access to testing, treatment, and effective antiviral therapy in this vulnerable population of the Brazilian Amazon.

## 1. Introduction

The hepatitis C virus (HCV), a positive-sense single-stranded RNA virus of the Flaviviridae family, represents a major global health burden [1]. This pathogen is characterized by considerable genetic diversity, comprising seven major genotypes and over 67 subtypes, a feature that significantly influences its geographical distribution and therapeutic outcomes [2]. Globally, an estimated 58 million people live with chronic HCV infection, though the majority remain asymptomatic. Moreover, the annual incidence of approximately 1.5 million new infections underscores the virus’s persistent transmission [3,4]. Within Brazil, an estimated 657,000 individuals are infected, with a prevalence dominated by genotypes 1, 2, and 3 [5,6].

Among Brazil’s diverse regions, the Amazon presents a particularly critical and challenging epidemiological setting. Characterized by relative isolation, socio-ecological diversity, and limited public health infrastructure, this region reports an HCV seroprevalence of 1–3% in the general population [5,7]. Notably, this burden is disproportionately concentrated in key vulnerable populations, such as people who use illicit drugs (PWUIDs) (23.1%) [8], female sex workers (FSWs) (10.7%) [7], and hemodialysis patients (8.4%) [9]. While injection drug use remains the most efficient route of HCV transmission, a growing body of evidence indicates that non-injecting drug use, particularly the smoking of crack cocaine—a solid, smokable form of cocaine that is highly addictive and associated with a public health epidemic—also constitutes a significant risk. This is primarily attributed to the sharing of crack cocaine pipes and straws, which can facilitate the transmission of blood-borne viruses through exposure to contaminated secretions and micro-hemorrhages in the oral and nasal mucosa [10,11].

Consequently, people who use non-injectable drugs represent a key population for HCV acquisition and ongoing transmission [1]. This transmission is further amplified by a syndemic of drug use and unprotected sexual intercourse, particularly in the context of sex work, as demonstrated in studies involving sex workers and men who have sex with men [5,7]. Besides that, the abuse of crack cocaine has emerged as a severe public health crisis, with an estimated 370,000 users nationwide and an HCV prevalence of 2.63% among people who use crack cocaine (PWUCC) [12,13].

The management of HCV has been revolutionized by direct-acting antiviral (DAA) regimens, which target viral non-structural proteins to potently inhibit replication across all genotypes [14]. Despite their high efficacy, pre-existing resistance-associated substitutions (RAS)—present in 2–29% of treatment-naive individuals—pose a threat to treatment success [15]. Specifically, these polymorphisms can hinder the achievement of a sustained virological response (SVR) [16,17]. In Brazil, the Public Health System (SUS) provides access to a range of DAAs, including daclatasvir, sofosbuvir, and glecaprevir/pibrentasvir, which are sometimes used in combination with older agents like ribavirin, guided by national clinical protocols [18].

The convergence of the high prevalence of HCV among people who use illicit drugs, the expansion of the crack cocaine epidemic, the presence of significant barriers to diagnosis and treatment in the Amazon, and the emergent threat of resistance to publicly funded DAAs collectively necessitates a detailed understanding of the circulating virus. Therefore, this study aimed to genetically and epidemiologically characterize HCV in a sample of PWUCC from the Brazilian Amazon. As the first report from the Brazilian Amazon to integrate HCV genotyping, RAS screening, and social data among PWUCC, this study bridges a critical gap between epidemiology and viral genomics. The inclusion of resistance mutations in this underserved cohort provides a distinctive perspective compared to other studies of PWUIDs in Brazil and Latin America. Collectively, our findings provide a robust evidence base to inform targeted public health strategies for the control and elimination of this hepatotropic virus.

## 2. Materials and Methods

### 2.1. Origin of Data and Biological Samples

This cross-sectional study was conducted with biological samples and sociodemographic data from 165 PWUCC. The participants were originally enrolled in an epidemiological study on Hepatitis E virus infection conducted in the municipality of Bragança, Pará, located in the Brazilian Amazon region [19]. The inclusion criteria were (1) self-reported crack cocaine use within the preceding three months; (2) age of 18 years or older; (3) not being under the influence of psychotropic substances during the research team interaction; and (4) provision of informed consent prior to initiation of the study protocol, which included providing a biological sample and completing the epidemiological assessment. Analysis of drug use patterns from this cohort revealed intensive consumption, with an average duration of crack cocaine use of 39.6 months (±20.5 months) and a mean daily consumption of 13.5 “rocks” (±7.5 stones). Community-based recruitment was carried out from March 2016 to October 2018 using the snowball sampling technique [8].

### 2.2. Viral RNA Detection

Viral RNA was isolated from plasma samples using the QIAmp Viral RNA Mini kit (Qiagen, Hilden, Germany). Subsequent cDNA synthesis was performed with the High-Capacity cDNA Reverse Transcription Kit (Applied Biosystems, Foster City, CA, USA). Real-time reverse transcription polymerase chain reaction (RT-PCR) was used to detect HCV RNA. It targeted a part of the virus’s 5′ untranslated region (UTR) and used the TaqMan Universal PCR Master Mix (Applied Biosystems, Foster City, CA, USA) [20]. The RT-PCR assay had a detection limit of 20 IU/mL. All laboratory procedures were performed in accordance with the manufacturers’ protocols, including positive and negative control samples in each run to ensure accuracy.

### 2.3. Genotyping and Resistance-Associated Substitution Analysis

Samples positive for HCV RNA were subjected to nested PCR amplification of partial NS5A and NS5B regions using primers and cycling conditions as previously described [21]. The resulting PCR amplicons were purified with the QIAquick PCR Purification Kit (QIAGEN, Hilden, Germany) and sequenced bidirectionally using the BigDye Terminator v3.1 Cycle Sequencing Kit (Applied Biosystems, Foster City, CA, USA). The chromatograms of the bidirectional sequences were evaluated and used to generate sequences of the samples using the AliView software, version 1.30 [22]. HCV genotypes and subtypes were determined using the online COMET HCV tool (https://comet.ucsd.edu/), accessed on 25 September 2023 [23].

The identification of RAS and predictions of phenotypic resistance to various direct-acting antiviral agents were performed using the online platforms geno2pheno [hcv] (https://hcv.geno2pheno.org; accessed on 2 October 2023) and HCV-GLUE (http://hcv-glue.cvr.gla.ac.uk; accessed on 2 October 2023). These tools employ distinct methodological approaches. Geno2pheno [hcv] applies a machine learning-based statistical framework to generate a quantitative resistance prediction. Conversely, HCV-GLUE functions as a sequence annotation system that integrates a curated database of known resistance mutations from published literature and clinical studies, assigning resistance based on the detection of established polymorphisms. The use of both platforms provided a complementary analysis, enabling cross-validation of the findings through independent methodological principles and thereby reinforcing the robustness of the resistance profile assessment.

### 2.4. Statistical Analysis

Sociodemographic and behavioral data were compiled in a Microsoft Excel database (Microsoft Corp., Redmond, WA, USA) and imported into STATA 18 for statistical analysis. Descriptive statistics were used to characterize the participant cohort. For viral subtype frequencies, 95% confidence intervals were calculated. All analyses were performed using STATA 18.

## 3. Results

### 3.1. Characteristics of PWUCC

The socio-demographic and -economic characteristics reported by most PWUCC were male, young (18–29 years old), self-identified as non-white, single, and heterosexual, with low educational attainment and low monthly income (see Table 1). Two other defining features of the PWUCC sample are worth highlighting: The majority of PWUCC reported not having access to public health services (91.5%) in the past 12 months, and more than half (57.0%) reported living in unstable housing conditions during the same period.

### 3.2. Prevalence of HCV RNA and Viral Subtypes

HCV RNA was detected in 22 (13.3%) of the PWUCC. Among those with HCV RNA, genotypes 1 (68.2%) and 3 (31.8%) were identified, further including the subtypes 1a (27.3%), 1b (40.9%), and 3a (31.8%) (see Table 2).

### 3.3. Resistance-Associated Substitutions

The frequency of RAS in the NS5A gene fragment was 18.1% (4/22) for daclatasvir. Among these, 4.5% (1/22) showed likely resistance to both daclatasvir and ombitasvir. The identified RAS included F/L37L (4/22, 18.1%), F/L37I (1/22, 4.5%), P58S (1/22, 4.5%), and F/L37L+H/Q54H (1/22, 4.5%). Furthermore, 9.0% (2/22) of the nucleotide sequences harbored more than one RAS (see Table 3). All RAS in the NS5A fragment were found among PWUCC infected with HCV subtype 1b. Conversely, all samples (22/22, 100%) presented at least three polymorphisms (not associated with protease inhibitor resistance) in the NS5A fragment, as analyzed by HCV-GLUE and geno2pheno [hcv] (Supplementary Materials—Table S1).

In addition, the frequency of RAS in the NS5B gene fragment was 13.6% (3/22) in the samples, with 9.0% (2/22) also showing resistance polymorphisms to dasabuvir. These RAS were found among PWUCC infected with HCV subtypes 1b and 3a. The mutations identified were F/L159F (1/22, 4.5%), F/L159F+C/N316N (1/22, 4.5%), C/N316N (2/22, 9.0%), and A/T/V150V (3/22, 13.6%) (see Table 4). Analysis of the NS5B gene fragment using the HCV-GLUE tool indicated that 18.1% (4/22) of sequences contained a polymorphism. In contrast, analysis of the same fragment using geno2pheno [hcv] indicated the presence of at least four polymorphisms in all samples (22/22, 100%) (Supplementary Materials—Table S2). Coverage data for the NS5B region were also derived from the geno2pheno analysis.

## 4. Discussion

The sociodemographic and economic profile of the PWUCC sample in this study is consistent with other surveys of people who use illicit drugs in Brazil and globally, being composed predominantly of young, non-white, single, heterosexual men [8,12,13]. Low monthly income and educational attainment indicate significant socioeconomic vulnerability, which is closely associated with limited health awareness and reduced engagement in risk mitigation strategies. The high proportions of PWUCC who reported no access to public health services and unstable housing further highlight the health risks and social vulnerability commonly documented in this population [2,8,11,13,24].

The detected HCV RNA prevalence of 13.3% among PWUCC in the coastal Amazon region aligns with rates previously reported in northern Brazil [5,7,8]. The presence and distribution of HCV genotypes 1 and 3 further corroborate epidemiological patterns observed in the Brazilian Amazon and other regions worldwide [25,26,27]. Although genotype 1 is highly prevalent throughout Brazil, distinct geographic patterns exist, with genotype 2 being more frequent in Central-West Brazil and genotype 3 commonly detected in Southern Brazil [26,28]. Globally, genotype 1 predominates in the Americas, Europe, Australia, and parts of Asia, while genotype 3 is highly prevalent in India and Pakistan. Genotype 4 is concentrated in Egypt and sub-Saharan Central Africa, and genotypes 5 and 6 show significant frequencies in South Africa and Southeast Asia, respectively [1,14].

A previous study of PWUCC along an international drug trafficking route in Central-West Brazil detected HCV RNA in 83.9% (26/31) of anti-HCV positive samples, revealing genotypes 1a (73.9%), 1b (8.7%), and 3a (17.4%) [13]. Similarly, research involving users of both injected and non-injected illicit drugs in Cuiabá, Central Brazil, found that 80% (16/20) of anti-HCV-positive participants were HCV RNA-positive, with genotype 1 predominating (75%), followed by 3a (25%). In that cohort, subtype 1a was more prevalent than 1b, and HCV infection was substantially more common among intravenous drug users (33%) than non-injecting users (1.5%) [29]. In the Amazon region, a study of FSWs showed HCV exposure in 10.7% (44/412) of participants and active infection in 7.8% (32/412), with genotypes 1 (81.3%) and 3 (18.7%) detected. Subtype analysis identified both 1a (34.4%) and 1b (46.9%) variants [7].

In our study, we identified HCV subtypes 1b (40.9%), 3a (31.8%), and 1a (27.3%). The substantial presence of subtype 3a, which is strongly associated with illicit drug use—particularly injection drug use—reflects the specific risk behaviors of the studied population [25,28]. This subtype is linked to more aggressive hepatitis C progression and a higher risk of hepatocellular carcinoma, even in the DAA era. Meanwhile, subtype 1b, the most prevalent in our sample, is frequently associated with treatment resistance and has historically shown poorer response to interferon-based therapies [1,25]. Together with data from the FSW study [7], our findings suggest a higher prevalence of subtype 1b in the Amazon region compared to studies of people who use illicit drugs in central Brazil [13,29].

This study also provides the first description of RAS and potential phenotypic resistance to DAAs among HCV-infected PWUCC in the Brazilian Amazon region. Our analysis revealed that 18.1% (4/22) of participants had RAS conferring resistance to daclatasvir in the NS5A region, with 4.5% (1/22) showing likely cross-resistance to ombitasvir. All NS5A RAS were exclusively identified in participants infected with subtype 1b. The F/L37L substitution (4/22, 18.1%) was the most frequent daclatasvir RAS, while F/L37L and P58S were associated with probable ombitasvir resistance. The strong association between subtype 1b and treatment resistance supports evidence that NS5A inhibitor-resistant variants can persist for years, if not indefinitely [25,30]. In the NS5B region, all participants with detectable RAS (5/22, 22.7%) exhibited potential resistance to sofosbuvir, with 9.0% (2/22) showing concurrent resistance to dasabuvir. The mutations F/L159F, C/N316N, and A/T/V150V were identified [31,32].

Regarding resistance patterns, evidence indicates that NS5A RAS can persist for over two years after DAA treatment. The loss of Y93H has been linked to diminished resistance in genotype 1a and 3 infections, whereas for genotype 1b, the NS5B RAS combination C316N + S556G appears to maintain stable persistence [33]. Supporting this, RAS analysis in FSWs demonstrated resistance to grazoprevir in 23.1% (6/26) of cases, associated with Y56F and S122G substitutions in NS5B [7]. Notably, our data confirm the presence of C316N in NS5B among genotype 1b-infected patients, underscoring the concerning epidemiological scenario where the most prevalent subtype also carries significant resistance potential.

The investigation of other polymorphisms revealed substantial viral variability. At least three mutations were detected in the NS5A region across all samples, while NS5B analysis indicated polymorphisms in 18.1% of samples using HCV-GLUE and 100% using geno2pheno. This genetic plasticity is characteristic of HCV, driven by its high replication rate and error-prone RNA-dependent RNA polymerase [14,34]. These findings should be interpreted within the context of the Brazilian Amazon as a region marked by significant health disparities, including high poverty rates, limited infrastructure, and inadequate health services, all of which facilitate infectious disease transmission [7,8,20,35].

Data showing that 91.5% (151/165) of participants had not accessed any health services in the twelve months prior to the study indicate that current healthcare strategies are failing to effectively reach this vulnerable population. The homelessness and drug use patterns characteristic of some PWUCC suggest that the “Consultório na Rua” program—an itinerant healthcare initiative within Brazil’s Unified Health System (SUS) employing multidisciplinary teams—could potentially engage this population [36,37]. Although this program operates in the study region, it had not reached these participants at the time of data collection.

Within SUS, clinical care follows standardized protocols and depends on medication availability. Ministry of Health guidelines determine treatment regimens based on genotype, liver disease stage, and treatment history, while also considering RAS [37]. When specific mutations are detected, protocols may recommend alternative therapies or extended treatment, creating a logistical requirement for resistance testing that is particularly challenging in Amazonian regions where test availability remains limited [38].

In resource-limited settings, medical decisions are often made without resistance data due to the cost-ineffectiveness of universal RAS testing. This creates a paradoxical situation where the constitutional guarantee of equitable healthcare conflicts with practical budget constraints [39,40]. For patients receiving ineffective empirical regimens, this testing gap can lead to treatment failure with serious individual health consequences and potential public health implications through continued transmission. Furthermore, the absence of reliable resistance data undermines monitoring efforts essential for evaluating health policy effectiveness.

The second-generation pan-genotypic regimen voxilaprevir (VOX)/velpatasvir (VEL)/sofosbuvir (SOF) demonstrates excellent efficacy, achieving SVR rates exceeding 95% regardless of RAS status, though reduced effectiveness is observed in genotype 3 patients with cirrhosis [33]. While this regimen has received SUS approval [41], regulatory clearance alone cannot guarantee access for PWUCC in the Amazon region, who require specifically tailored approaches to ensure appropriate diagnosis and treatment. Similar implementation challenges affect other pan-genotypic regimens like VEL/SOF and glecaprevir/pibrentasvir.

Overall, these results emphasize the critical need to enhance HCV diagnosis and treatment for PWUCC by incorporating advanced genotyping and RAS assessment into clinical practice. The identified genetic profiles should alert public health authorities and reinforce the urgency of implementing measures to curb HCV transmission. Furthermore, since limited healthcare access and poor treatment adherence increase the likelihood of resistant mutations, educational and harm-reduction policies should be implemented to promote safer sexual and drug-use practices among at-risk individuals [12,42]. Strategies such as the “Consultório na Rua” program represent crucial resources for providing psychosocial support and rapid testing for STIs/HIV, serving as a bridge between health services and PWUCC [37]. Ultimately, recognizing PWUCC as full members of Brazilian society with equal rights to healthcare is essential. A care model based on co-responsibility, supporting individuals from diagnosis through treatment completion, is vital for achieving comprehensive and effective health outcomes [42,43].

This study has several limitations that should be acknowledged. The PWUCC sample consisted of a small convenience sample and, therefore, is not generalizable to other populations of PWUCC or individuals who use illicit drugs. Furthermore, epidemiological factors were recorded based on self-reporting, and there was no control for behavioral confounders, which renders these data not objectively verifiable and potentially subject to bias. Genotypic resistance testing also identified drug resistance; however, the analysis of the NS5B region is limited by insufficient sequencing coverage, which precludes a more definitive result. Minor genetic variants of HCV can be detected with greater sensitivity using next-generation sequencing; this is essential for greater accuracy in genotyping and monitoring of RAS. Additionally, the cross-sectional design precludes causal inference, and the sample size is insufficient for a robust inferential analysis of resistance. Longitudinal studies or phylogenetic tracking represent viable approaches to mitigate these limitations in future research.

## 5. Conclusions

This study identified critical genetic characteristics of HCV present among PWUCC in the Brazilian Amazon, a region featuring limited health infrastructure. The high prevalence of HCV infection within this vulnerable and marginalized population, coupled with the predominance of subtypes 1b and 3a and the presence of RAS, underscores the need to implement targeted HCV prevention and care strategies. Such strategies must guarantee equitable access to diagnosis and treatment for PWUCC and the general population. Failure by local and regional health authorities to address this situation more effectively risks a severe and growing public health crisis in the future. Without robust surveillance, diagnostic, and treatment measures, the silent dissemination of HCV strains with amplified treatment resistance and a potential for progression to cirrhosis and hepatocellular carcinoma is likely to continue unabated. Consequently, a follow-up study to track the temporal trends in HCV prevalence within this population will be of critical importance for guiding future public health interventions.

## Figures and Tables

**Table 1:** Demographic and socioeconomic characteristics of PWUCC in the municipality of Bragança in the Brazilian Amazon region.

Characteristics	N (%)
Total	165 (100.0)
Sex	
Male	116 (70.3)
Female	49 (29.7)
Age (years)	
18–29	102 (61.8)
30–40	56 (33.9)
>40	7 (4.3)
Color/Race (self-declaration)	
White	22 (13.3)
Brown (mixed race)	113 (68.5)
Black	30 (18.2)
Marital status	
Single	134 (81.2)
Separated or widowed	22 (13.3)
Married or co-habitating	9 (5.5)
Education Level	
No formal education (including illiterates)	10 (6.1)
Elementary school	113 (68.4)
High school	32 (19.4)
University	10 (6.1)
Monthly income (minimum wage) ^†^	
≤1 ^‡^	119 (72.1)
2–3	39 (23.6)
>3	7 (4.3)
Sexual orientation	
Heterosexual	150 (90.9)
Homosexual	9 (5.5)
Bisexual	6 (3.6)
Housing status ^†^	
Lives in his own house/with his parents	71 (43.0)
Lives in a house or rented room	63 (38.2)
Unstable housing (including homeless people)	31 (18.8)
Access to public health service	
Yes	14 (8.5)
No	151 (91.5)

^†^ Last 12 months; ^‡^ One minimum wage = R$ 945 (equivalent to US$ 190).

**Table 2:** Prevalence of HCV genetic markers among PWUCC in the municipality of Bragança in the Brazilian Amazon region.

Marker	Positive/Total	Prevalence (95% CI)
HCV RNA	22/165	13.3 (9.2–17.1)
Genotype 1	15/22	68.2 (63.4–73.4)
Subtype 1a	6/22	27.3 (22.5–32.6)
Subtype 1b	9/22	40.9 (35.7–45.7)
Genotype 3	7/22	31.8 (27.3–36.9)
Subtype 3a	7/22	31.8 (27.3–36.9)

**Table 3:** Description and frequencies of subtypes, RAS and probable resistance identified from HCV NS5A detected among PWUCC in the municipality of Bragança in the Brazilian Amazon region.

Sample	Subtype	Coverage (%)	RAS	Probable Resistance *
PWUCC01	1a	96.2	-	None
PWUCC02	1a	96.2	-	None
PWUCC03	1a	96.7	-	None
PWUCC04	1a	98.4	-	None
PWUCC05	1a	99.6	-	None
PWUCC06	1a	96.7	-	None
PWUCC07	1b	36.2	-	None
PWUCC08	1b	26.1	F/L37L	Daclatasvir
PWUCC09	1b	35.5	F/L37I	Daclatasvir
PWUCC10	1b	60.5	-	None
PWUCC11	1b	68.8	F/L37L, P58S	Daclatasvir and Ombitasvir
PWUCC12 PWUCC13 PWUCC14 PWUCC15 PWUCC16 PWUCC17 PWUCC18 PWUCC19 PWUCC20 PWUCC21 PWUCC22	1b 1b 1b 1b 3a 3a 3a 3a 3a 3a 3a	88.6 86.3 88.6 88.6 67.4 99.1 52.7 71.7 99.8 71.0 99.8	F/L37L+H/Q54H - - F/L37L - - - - - - -	Daclatasvir None None Daclatasvir None None None None None None None
Total frequency				5/22 (22.7%)
Daclatasvir (F/L37L, F/L37I or F/L37L+H/Q54H)				4/22 (18.1%)
Daclatasvir and Ombitasvir (F/L37L and P58S)				1/22 (4.5%)

* None: No probable resistance detected.

**Table 4:** Description and frequencies of subtypes, RAS and probable resistance identified from HCV NS5B detected among PWUCC in the municipality of Bragança in the Brazilian Amazon region.

Sample	Subtype	Coverage (%)	RAS	Probable Resistance *
PWUCC01	1a	94.89	-	None
PWUCC02	1a	96.56	-	None
PWUCC03	1a	93.41	-	None
PWUCC04	1a	95.48	-	None
PWUCC05	1a	95.28	-	None
PWUCC06	1a	95.87	-	None
PWUCC07	1b	93.62	-	None
PWUCC08	1b	94.01	-	None
PWUCC09	1b	93.04	F/L159F, F/L159F+C/N316N, C/N316N	Dasabuvir and Sofosbuvir
PWUCC10	1b	93.73	-	None
PWUCC11	1b	93.5	C/N316N	Dasabuvir and Sofosbuvir
PWUCC12 PWUCC13 PWUCC14 PWUCC15 PWUCC16 PWUCC17 PWUCC18 PWUCC19 PWUCC20 PWUCC21 PWUCC22	1b 1b 1b 1b 3a 3a 3a 3a 3a 3a 3a	92.82 94.59 93.62 93.84 95.46 96.22 94.71 96.07 94.48 94.94 95.01	- - - - - - - A/T/V150V A/T/V150V - A/T/V150V	None None None None None None None Sofosbuvir Sofosbuvir None Sofosbuvir
Total frequency				5/22 (22.7%)
Dasabuvir and sofosbuvir (F/L159F, F/L159F+C/N316N and/or C/N316N)				2/22 (9.0%)
Sofosbuvir (A/T/V150V)				3/22 (13.6%)

* None: No probable resistance detected.

## Data Availability

The HCV sequences from this study were deposited in GenBank under accession numbers PX468912—PX468933 (NS5A) and PX468934—PX468955 (NS5B). Other data analyzed during the current study are not publicly available due to ongoing analyses of possible infections and co-infections with other pathogens but may be made available by the corresponding author upon reasonable request.

## Supplementary Materials

The following supporting information can be downloaded at: https://www.mdpi.com/article/10.3390/pathogens14121296/s1, Table S1: Description of HCV subtypes and mutations found in NS5A of the virus detected among PWUCC in the municipality of Bragança in the Brazilian Amazon region; Table S2: Description of HCV subtypes and mutations found in NS5B of the virus detected among PWUCC in the municipality of Bragança in the Brazilian Amazon region.

## Abbreviations

The following abbreviations are used in this manuscript:

DAAs	Direct-acting antiviral
FSWs	Female sex workers
PWUIDs	People who use illicit drugs
HCV	Hepatitis C virus
PWUCC	People who use crack cocaine
RAS	Resistance-associated substitutions
RT-PCR	Real-time reverse transcription polymerase chain reaction
SOF	Sofosbuvir
SUS	Brazil’s Public Health System
SVR	Sustained virological response
UTR	5′ untranslated region
VEL	Velpatasvir
VOX	Voxilaprevir
