# Supplementary material for: Genetic Characterization of Hepatitis C Virus Among People Who Use Crack Cocaine: A Study Conducted on the Brazilian Amazon Coast
Source: Pathogens. 2025 Dec 17;14(12):1296. doi: 10.3390/pathogens14121296 (PMC12735445; doi:10.3390/pathogens14121296)
Supplement: Supplementary file 1 [file pathogens-14-01296-s001.zip › pathogens-4024354-supplementary.pdf]

SUPPLEMENTARY MATERIALS

Title: Genetic Characterization of Hepatitis C Virus Among People Who Use Crack-cocaine: a Study Conducted on the Brazilian Amazon Coast.

Authors: João Alphonse A. Heymbeeck, Wilker Leite do Nascimento, Marina Cristina S. Freitas, Leticia de Sousa Rocha, Franciane Ferreira Costa, Jocilena Pamela Q. de Queiroz, Diego Simeone, Luísa Caricio Martins, Luiz Fernando A. Machado, Benedikt Fischer, Emil Kupek and Aldemir B. Oliveira-Filho.

Table S1: Description of HCV subtypes and mutations found in NS5A of the virus detected among PWUCC in the municipality of Bragança in the Brazilian Amazon region.

Samples	Subtypes	Other mutations (HCV-GLUE)	Other mutations (geno2pheno)
PWUCC01	1a	K24K+M28M, K24K+M28M+Q30Q, K24K+M28M+Q30Q+V37V+ H54H, M28M, Q30Q	D7E, F36L, R48Q, R78K, K107E, S131T, I144V, F161Y, A213T
PWUCC02	1a	K24K+M28M, K24K+M28M+Q30Q, K24K+M28M+Q30Q+V37V+ H54H, M28M, Q30Q	F36L, R78K, R123Q, S131T, I144V, L158I, A213T
PWUCC03	1a	K24K+M28M, K24K+M28M+Q30Q, K24K+M28M+Q30Q+V37V+ H54H, M28M, Q30Q, H58N	F36L, H58N, R78K, T87S, I121V, R123Q, S131T, I144V, A213T
PWUCC04	1a	K24K+M28M, K24K+M28M+Q30Q, K24K+M28M+Q30Q+V37V+ H54H, M28M, Q30Q	F36L, R78K, R123Q, S131T, I144V, A197T, A213T
PWUCC05	1a	K24K+M28M, K24K+M28M+Q30Q, K24K+M28M+Q30Q+V37V+ H54H, M28M, Q30Q, H58P	F36L, H58P, R78K, W111L, I121V, R123Q, S131T, M133V, I144V, F161Y, E171D, S174T, Y182F, A197S, A213T
PWUCC06	1a	K24K+M28M, K24K+M28M+Q30Q, K24K+M28M+Q30Q+V37V+ H54H, M28M, Q30Q	R78K, R123Q, S131T, I144V, E181D, A213T
PWUCC07	1b	R30R, A92T, L28L, P58P	K6R, W9R, S17T, L34V, L37F, K44R, T83M, A92T, V138L
PWUCC08	1b	R30R, L28L, P58P	K6R, S17T, L34V, T64A, P104L, N105I, R108K
PWUCC09	1b	R30R, L28L, P58P, T56I	K6R, L34V, L37I, T56I, T64A, K78R

PWUCC10	1b	R30R, L28L, P58P, T56I, T99S	K6R, S17T, L34V, L37F, Q54H, T56I, C98S, T99S, R108K, V138I, V164A, E171D, V174T, L183V, A197T, S207A
PWUCC11	1b	R30R, L28L, Y129F	K6R, S17T, K26R, L27I, L34I, P58S, T64A, T122V, F127S, Y129F, M133V, V164A, E171D, V174T, A197T
PWUCC12	1b	R30R, L28L, P58P	K6R, S17T, L34I, F36L, Q54H, V121I, V138I, V164A, E171D, L183P, A197V, L199V
PWUCC13	1b	K24K+M28M, K24K+M28M+Q30Q, K24K+M28M+Q30Q+V37V+ H54H, M28M, Q30Q	A61D, K107T, W111L, I121V, R122K, S131T, I144V, F161Y, E171D, A197S
PWUCC14	1b	K24K+M28M, K24K+M28M+Q30Q, K24K+M28M+Q30Q+V37V+ H54H, M28M, Q30Q, H58P	R44K, R48Q, H58P, R78K, K107E, R123Q, S131T, I144V, F161Y, A213T
PWUCC15	1b	R30R, L28L, P58P	K6R, S17T, L34V, K44R, T64A, S107F, R108K, R123Q, M133V, V138I, V164A, V174T, A197T, T204S, S207A
PWUCC16	3a	S24S, M28M, A30A, I37I, P58P	T7D, S14T, A17S, A21T, A62T, H85Y, S98G, N116S, D172E, M176T, H180N, T183A, S197C, V198A
PWUCC17	3a	S24S, A30A, I37I, P58P, M28R, A75V	T7D, S14T, A17N, A21T, A25G, M28R, A62S, T64A, A75V, H85Y, D172E, H180N, T183A, H208Q
PWUCC18	3a	S24S, M28M, A30A, I37I, P58P, L23I	T7D, S14T, A17Q, A21T, L23I, A25T, A62S, T64S, N116S, E137G, H180N, T183V
PWUCC19	3a	S24S, M28M, A30A, I37I, P58P, A30V	T7D, S14T, A17S, A21T, A30V, A62S, S103P, M176T, H180N, T183V
PWUCC20	3a	S24S, M28M, A30A, I37I, P58P	T7D, S14T, A17C, A21R, A62T, T64S, V124M, L158I,

			Y161F, D172E, H180N, T183A
PWUCC21	3a	S24S, M28M, A30A, I37I, P58P	T7D, S14T, A17Q, A21T, A62S, T64S, H85Y, S98G, L158I, D172E, H180N, T183A
PWUCC22	3a	S24S, M28M, A30A, I37I, P58P	T7D, S14T, A17S, A21T, A62S, S98G, H180N, T183V
**Total Frequency**		**22/22 (100%)**	**22/22 (100%)**

Table S2: Description of HCV subtypes and mutations found in NS5B of the virus detected among PWUCC in the municipality of Bragança in the Brazilian Amazon region.

Sample	Subtypes	Other mutations (HCV-GLUE)	Other mutations (geno2pheno)
PWUCC01	1a	-	V11I, L36M, S62N, Q65R, K98R, A117N, S130N, S180T, L184P, R300Q, Q309R, A327E
PWUCC02	1a	-	V11I, K98R, A117N, V178L
PWUCC03	1a	E150A	S3R, S5Y, W6Q, V11I, K98R, A117N, S130N, Q148E, E150A, V178L, L184I, S189N, G198A, K212R, S231N, N273S, R300Q, Q309R, A327V
PWUCC04	1a	-	V11I, C46S, K98R, A117N, S231N, R300Q
PWUCC05	1a	-	V11I, C46S, E87D, K98R, A117N, R300Q, Q309L
PWUCC06	1a	-	V11I, H63Y, K98R, A117N, K212R, S231N, R300Q, Q309R
PWUCC07	1b	-	M57L, K81R, Q90K, R98K, R120N, Q127L, E131D, D135E, T136A, V147I, V235T, I262V
PWUCC08	1b	-	V37I, M57L, K81R, Q90K, R98K, K114R, N117D, Q127L, T130S, T181N, S300T, V338A
PWUCC09	1b	-	I23V, M57L, S84P, Q90R, Q127L, E131D, L159F,

			C213S, A218S, S231N, C316N, S335N
PWUCC10	1b	-	M57L, M71V, Q90K, Q127L, E131D, S189P, R222P, S231N, I262V, S335N
PWUCC11	1b	-	M57L, Q90K, R98K, Q127L, E131D, K212R, C213S, A218S, S231N, I262V, D310G, C316N
PWUCC12	1b	-	M57L, M71V, Q90K, Q127L, E131D, S189P, R222P, S231N, S300T, Q309K
PWUCC13	1b	-	M57L, Q90K, S113R, N117D, N206K, K209T, I262V, S300T
PWUCC14	1b	-	M57L, R65Q, E87D, Q90K, K114R, V116L, K124E, Q127L, C213N, S231N, V235T, K270R
PWUCC15	1b	-	M57L, M71V, Q90K, Q127L, E131D, S189P, R222P
PWUCC16	3a	E/K206Q	K50R, R77K, L111M, R114K, P189S, K206Q, K304R, N307G, R333K
PWUCC17	3a	E/K206Q	K50R, R77K, L111M, R114K, P189S, K206Q, I233V, N307G, R337G, A356P, R379K, M426LM
PWUCC18	3a	-	V71I, R77K, V108A, R114K, I116V, V169I, P189S, S254T, N307G, A356P, R379K, A393V
PWUCC19	3a	-	R77K, L111F, R114K, A150V, P189S, K211R, N244D, K304R, N307G, A356P, R379K
PUCC20	3a	E/K206Q	R114K, A150V, P189S, K206Q, N244D, K304R, N307G, A356P, R379K
PWUCC21	3a	-	Q47H, A67V, V71I, R77K, S104G, P189S, N244D, N307G, R337K, A356P, R379K, A393V

PWUCC22	3a	-	R77K, S104G, R114K, A150V, N244D, N307G, D327E, D330N, R337G, R374L, R379K
**Total Frequency**		**4/22 (18.1%)**	**22/22 (100%)**
